# Polymyxin B Hemoperfusion for Patients With Septic Shock Requiring High-Dose Norepinephrine: A Multicenter Prospective Cohort Study

**DOI:** 10.1097/CCE.0000000000001320

**Published:** 2025-09-22

**Authors:** Yu Kawazoe, Kyohei Miyamoto, Noriko Miyagawa, Yoshinori Ohta, Hitoshi Yamamura, Takuya Kimura, Yukitoshi Toyoda, Michihito Kyo, Tetsuya Sato, Masashi Kinjo, Masaki Takahashi, Junichi Maruyama, Kazunori Fukushima, Hiroshi Matsuura, Satoru Murata, Tomoya Okazaki, Toshihiro Sakurai, Tsuyoshi Suzuki, Tasuku Hanajima, Gaku Takahashi, Takeshi Morimoto

**Affiliations:** 1 Department of Emergency and Critical Care Medicine, Tohoku University Hospital, Sendai, Japan.; 2 Department of Emergency and Critical Care Medicine, Wakayama Medical University, Wakayama, Japan.; 3 Department of Emergency and Critical Care Medicine, Sendai Medical Center, Sendai, Japan.; 4 Department of Emergency and Critical Care Medicine, National Hospital Organization Kyoto Medical Center, Kyoto, Japan.; 5 Department of Emergency and Critical Care Medicine, Japan Community Health Care Organization, Osaka Minato Central Hospital, Osaka, Japan.; 6 Department of Emergency Medicine and Critical Care Medicine, Saiseikai Utsunomiya Hospital, Utsunomiya, Japan.; 7 Department of Emergency and Critical Care Medicine, Hiratsuka City Hospital, Hiratsuka, Kanagawa, Japan.; 8 Department of Emergency and Critical Care Medicine, Graduate School of Biomedical and Health Sciences, Hiroshima University, Hiroshima, Japan.; 9 Division of Critical Care Medicine, Nara Prefecture General Medical Center, Nara, Japan.; 10 Division of Acute and Critical Care Medicine, Department of Anesthesiology and Critical Care Medicine, Hokkaido University Faculty of Medicine, Sapporo, Japan.; 11 Department of Emergency Medicine and Critical Care, Fukuoka University Hospital, Fukuoka, Japan.; 12 Department of Emergency Medicine, Gunma University Graduate School of Medicine, Maebashi, Gunma, Japan.; 13 Department of Emergency and Critical Care Medicine, Osaka Prefectural Nakakawachi Emergency and Critical Care Center, Higashi-Osaka, Japan.; 14 Department of Emergency and Critical Care Medicine, Ehime University Graduate School of Medicine, Toon, Japan.; 15 Department of Emergency Medical Center, Kagawa University Hospital, Kida, Japan.; 16 Department of Emergency and Critical Care Medicine, National Hospital Organization Kumamoto Medical Center, Kumamoto, Japan.; 17 Department of Emergency and Critical Care Medicine, Fukushima Medical University, Fukushima, Japan.; 18 Department of Trauma and Reconstruction Center, Shin-yurigaoka General Hospital, Kawasaki, Japan.; 19 Department of Critical Care and Disaster Medicine, Iwate Medical University, Morioka, Japan.; 20 Department of Data Science, Hyogo Medical University, Nishinomiya, Japan.

**Keywords:** endotoxin, hemoperfusion, long duration, polymyxin B, septic shock

## Abstract

**IMPORTANCE:**

Polymyxin B hemoperfusion (PMX-HP) is a potential therapeutic option for sepsis; however, its clinical effects remain unclear.

**OBJECTIVE:**

We explored the association between PMX-HP and clinical outcomes in patients in ICUs.

**DESIGN, SETTING, AND PARTICIPANTS:**

This is a multicenter prospective cohort study conducted in 20 ICUs in Japan between 2020 and 2022. Patients with septic shock requiring high-dose norepinephrine (≥ 0.2 µg/kg/min) were included.

**MAIN OUTCOMES AND MEASURES:**

We compared patient characteristics and outcomes between those who received PMX-HP (PMX group) and those who did not (non-PMX group). A primary outcome was 28-day mortality, with secondary outcomes including changes in mean arterial pressure (MAP) and vasoactive-inotropic score over time in the initial 48 hours, 28-day vasopressor-free days, and 90-day mortality rate.

**RESULTS:**

Among 309 patients, 175 (56.6%) were males, with a median age of 72 years and an Acute Physiology and Chronic Health Evaluation II (APACHE II) score of 26. Overall, 82 patients underwent PMX-HP. The PMX and non-PMX groups had similar median ages (71 vs. 73 yr) and APACHE II scores (26 vs. 27). The median PMX-HP duration was 1016 minutes. The 28-day mortality was similar between the groups (PMX group: 17.1%, non-PMX group: 18.9%, *p* = 0.71), with an adjusted hazard ratio of 0.88 (95% CI, 0.46–1.68). MAP was maintained at a higher level with low-dose vasopressor from 8 to 32 hours following admission in the PMX group. The PMX group had fewer 28-day ICU-free days (16 vs. 18, *p* = 0.026). The 90-day mortality rate was similar between the groups (PMX group, 23.5%; non-PMX group, 27.1%; *p* = 0.62).

**CONCLUSIONS AND RELEVANCE:**

PMX-HP was not associated with the 28-day mortality improvement but was associated with higher MAP and lower vasopressor use for 8–32 hours of admission. Our findings suggested that PMX-HP requires careful adaptation.

**KEY POINTS**

**Question**: Polymyxin B hemoperfusion (PMX-HP) is a potential therapeutic option for sepsis; therefore, we explored the association between PMX-HP and clinical outcomes in patients with severe septic shock.

**Findings**: In our prospective cohort study, PMX-HP was not associated with 28-day/90-day mortality improvement. PMX-HP was associated with high mean arterial pressure with low vasopressor use for 8–32 hours of admission; however, the PMX group exhibited fewer 28-day ICU-free days than the non-PMX group.

**Meanings**: Our findings suggested that PMX-HP performed for an extended period may have more disadvantages than benefits, requiring careful adaptation.

Mortality rates for sepsis remain high, particularly for patients with septic shock (1–3). Various treatment strategies have been explored, including optimized fluid resuscitation, corticosteroids, vasopressor selection (including newer options), and investigational treatments, including extracorporeal therapies such as polymyxin B hemoperfusion (PMX-HP) and other cytokine adsorption therapies (4–7). PMX-HP was proposed as an adjunctive therapy for septic shock by removing circulating endotoxins (8, 9). Studies have linked PMX-HP to increased blood pressure and improved oxygenation (10, 11); however, randomized clinical trials have not shown survival benefits (10, 12, 13). Nevertheless, several Japanese reports suggest a survival benefit from PMX-HP in patients with septic shock (14–17) and many physicians continue to use PMX-HP in septic shock with insurance coverage. In Japan, some physicians clinically use PMX-HP for a longer duration than the global standard of 2 hours (18–20), especially in patients with severe septic shock.

To explore the clinical roles of PMX-HP for septic shock patients in terms of several potential markers as well as mortality in the real-world setting, we analyzed the data of a prospective cohort study as hypothesis-generating research.

## MATERIALS AND METHODS

This article reports the effects of PMX-HP on outcomes among patients registered in the BEst Available Treatment for septic SHOCK Registry (BEAT-SHOCK Registry).

### BEAT-SHOCK Registry

The BEAT-SHOCK Registry was a multicenter, prospective study of consecutive patients with septic shock who received intermediate and high doses of norepinephrine (≥ 0.2 µg/kg/min) (21). The sample size was determined to be 400 participants based on the expected number of patients during the study period. The registry was conducted across 20 ICUs in Japan from January 2020 to December 2022, and included adults aged 18 years old or older admitted to the ICU with septic shock requiring greater than or equal to 0.2 µg/kg/min of norepinephrine within 24 hours of sepsis diagnosis due to acute infection. The norepinephrine dose was recorded as the base-equivalent dose. Sepsis and septic shock were defined according to Sepsis-3 criteria (1).

The exclusion criteria comprised patients who were discharged from the ICU or died within 48 hours; those with a limited long-term prognosis due to malignancy, pre-existing organ damage, acute myocardial infarction, severe heart failure (New York Heart Association Class IV), or severe liver damage (Child-Pugh grade C); cases deemed undesirable by the physician; those who opted out, or patients with COVID-19 as the primary etiology. Patients who improved or died within 48 hours were excluded, as they were presumed to be unsuitable for evaluating the therapeutic effect of treatment, including PMX-HP, during the acute phase.

This study was approved by the ethics committee of Tohoku University Hospital on September 17, 2019 (Approval No. 2019-1-402) and all participating hospitals in accordance with the Ethical Guidelines for Medical and Biological Research Involving Human Subjects (22) and the principles of the Helsinki Declaration. This study was registered with the University Hospital Medical Information Network Clinical Trial Registry (UMIN000038302) before the commencement of the trial and enrollment of any patient. Informed consent was obtained through the opt-out method. Written informed consent was only required from patients or their representatives for the collection of outcome data at 90 days.

### Data Collection and Variables

All clinical data were registered by the physician-in-charge in an electronic data capture (EDC) system. These data were centrally monitored by the data managers of the Institute for Clinical Effectiveness, a nonprofit research organization, and investigators were not directly involved in data management. The Clinical Course Evaluation Committee reviewed and validated the clinical data for consistency within the EDC system. Any identified inconsistencies were clarified from the data center, which were resolved by the physician-in-charge. Data collection was completed in February 2024.

The registered variables included age, sex, weight, disease name, Acute Physiology and Chronic Health Evaluation II (APACHE II) score at admission, pre-existing conditions, activities of daily living before admission, long-term care facility or nursing home, ICU admission route, sepsis onset date and time, initial lactate measurement date and time, end of initial infusion, and norepinephrine administration start date and time. Collected outcomes included ICU discharge date; 7-day, 28-day, and 90-day outcomes; cause of death; chronic dialysis transition; ventilator management dates; emergent surgery details; blood culture data; PMX-HP duration and interruptions; anticoagulants used; kidney replacement therapy (KRT) details; vital signs and catecholamine doses (every 2 hr up to 48 hr, then daily up to 7 d); infusion doses; blood transfusion; urine output; laboratory data; body weight; enteral and IV nutrition details; sedative and antimicrobial drug type and doses; consciousness level (Glasgow Coma Scale); sedation depth (Richmond Agitation-Sedation Scale); delirium (Confusion Assessment Method in the ICU); rehabilitation status; acute respiratory distress syndrome onset and management; prone ventilation status; extracorporeal membrane oxygenation details.

Survival and functional outcomes (e.g., Barthel Index, Short Memory Questionnaire, Impact of Event Scale-Revised) were assessed at 90 days through questionnaires sent to patients or their family members.

### Outcomes

The primary outcome was 28-day mortality, whereas secondary outcomes included change in mean arterial pressure (MAP) and vasoactive-inotropic score (VIS) over the initial 48 hours, 28-day ICU-free days, ventilator-free days, vasopressor-free days, hemodialysis-free days, and 90-day mortality. VIS was calculated using the following equation:

VIS = norepinephrine (µg/kg/min) × 100 + epinephrine (µg/kg/min) × 100 + dopamine (µg/kg/min) + dobutamine (µg/kg/min) + vasopressin (unit/kg/min) × 10,000 + levosimendan (µg/kg/min) × 50 + milrinone (µg/kg/min) × 10 (23).

VIS was calculated using only norepinephrine, epinephrine, dopamine, dobutamine, and vasopressin, as no patients received levosimendan or milrinone. The Sequential Organ Failure Assessment (SOFA) score over 3 days was subtracted from the score on day 1 to assess changes in severity. Changes in VIS, lactate clearance, fluid infusion, and transfusion volume within 48 hours were examined. Changes in VIS were obtained by subtracting the maximum VIS in the initial 6 hours from the VIS at 48 hours. In Japan, PMX-HP was approved by the Pharmaceuticals and Medical Devices Agency as a treatment for septic shock presumed to be caused by gram-negative bacteria for greater than or equal to 2 hours. In Japan, the duration of PMX-HP is preferred to be greater than or equal to 2 hours compared with the conventional duration (2 hr); however, the indication and duration of PMX-HP remained at the discretion of the attending physicians.

As a post hoc analysis of adverse events, the association between thrombocytopenia and the implementation of PMX-HP and continuous kidney replacement therapy (CKRT) was analyzed.

### Statistical Analysis

We compared patient characteristics and outcomes between those who received PMX-HP (PMX group) and those who did not (non-PMX group).

Continuous variables are reported as medians and interquartile ranges (IQRs) and categorical variables as numbers and percentages (%). Continuous variables were compared using the Wilcoxon rank-sum test, and categorical variables were compared using the chi-square or Fisher exact test. The Wilcoxon rank-sum test was used for each comparison between the groups regarding the MAP and VIS over time. Kaplan-Meier estimates were used to calculate cumulative 90-day survival owing to several losses to the 90-day follow-up. Cox proportional hazard models were constructed to estimate hazard ratios (HRs) and 95% CIs for the PMX group relative to the non-PMX group. Multivariate models were created using predefined adjusters, including age (65 yr old or older), chronic illness (Charlson comorbidity index [CCI] ≥ 3), disability (performance status ≥ 3), illness severity (APACHE II score ≥ 21), infection source (urinary tract/abdomen or not), surgical/medical status, serum lactate concentration (≥ 4 mmol/L), VIS (≥ 30), and bacteremia (yes/no), to obtain adjusted HRs. Cox proportional hazard models were constructed in prespecified subgroups: age (65 yr old or older vs. 65 yr old or younger), APACHE Ⅱ score (≥ 21 vs. < 21), SOFA score (≥ 11 vs. < 11), respiratory failure, renal failure, disseminated intravascular coagulation (DIC), infection sites (urinary, abdomen, thorax), causative organisms (gram-negative, gram-positive), lactate level (≥ 4 vs. < 4 mmol/L), and VIS (≥ 30 vs. < 30) at ICU admission. These cutoffs were set using predetermined clinically meaningful values; however, for the SOFA score, we adopted the median of the cohort because an appropriate cutoff value was not available.

In a post hoc analysis of adverse events, the odds ratio was calculated using univariate and multivariate logistic regression models with PMX-HP and CKRT as covariates.

There were no missing data regarding the primary outcome and covariates used in the multivariate models. All reported *p* values were two-sided, and statistical significance was set at *p* value of less than 0.05. All analyses were performed using JMP Pro Software (Version 17.2; SAS Institute, Cary, NC).

## RESULTS

### Baseline Characteristics

**Figure 1.**
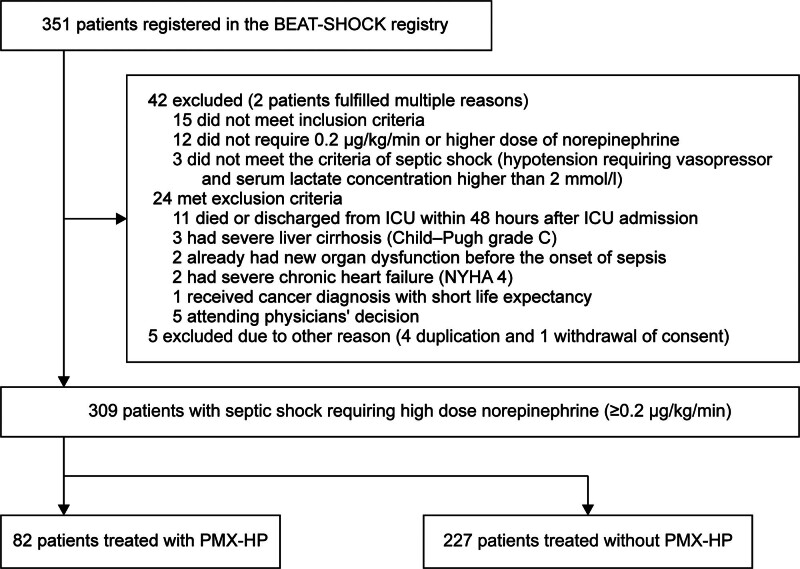
Patient selection flowchart. BEAT-SHOCK = BEst Available Treatment for septic SHOCK; NYHA = New York Heart Association classification for heart failure, PMX-HP = polymyxin B hemoperfusion

The BEAT-SHOCK Registry initially registered 351 patients, with 309 analyzed after excluding 42 patients (**Fig. 1**). There was no missing data on the primary outcome and adjusters used in multivariate models. **Table 1**; and **Supplementary Table 1** (https://links.lww.com/CCX/B554) include all patient characteristics, conditions, and treatments at admission. The median age was 72 years (IQR, 64–81), and 175 (56.6%) were males. The median APACHE II score and CCI were 26 (IQR, 21–33) and 1 (IQR, 0–3), respectively. The median SOFA score on the first day was 11 (IQR, 9–14). The median lactate concentration upon admission and maximum initial VIS at 6 hours post-admission were 4.1 mmol/L (IQR, 2.6–6.6) and 34.4 (IQR, 24.2–45.7), respectively. The 28-day mortality rate was 18.4%.

**TABLE 1.** Patient Characteristics Upon ICU Admission

Characteristics	All Patients (*n* = 309)	PMX Group (*n* = 82)	Non-PMX Group (*n* = 227)	*p*
Age (yr)	72 (64–80.5)	71 (62–80)	73 (65–81)	0.26
Male sex	175 (56.6)	46 (56.1)	129 (56.8)	0.91
Site of infection				0.031
Abdomen	115 (37.2)	37 (45.1)	78 (34.4)	
Urinary tract	50 (16.1)	18 (22.0)	32 (14.1)	
Thorax	59 (19.1)	8 (9.8)	51 (22.5)	
Skin and soft tissue	56 (18.1)	14 (17.1)	42 (18.5)	
Others	29 (9.4)	5 (6.1)	24 (10.6)	
Causative organisms				0.48
Gram-negative bacteria	135 (43.7)	39 (47.6)	96 (42.3)	
Gram-positive bacteria	50 (16.2)	10 (12.2)	40 (17.6)	
Others (including unknown)	124 (40.1)	33 (40.2)	91 (40.1)	
Acute Physiology and Chronic Health Evaluation II score	26 (21–32.5)	26 (21–31)	27 (22–33)	0.37
Sequential Organ Failure Assessment score	11 (9–14)	11 (9–13)	11 (9–14)	0.093
Emergency surgery^a^	108 (35.0)	36 (43.9)	72 (31.7)	0.047

APACHE II = Acute Physiology and Chronic Health Evaluation II, PMX = polymyxin B.

a Emergency surgery was defined as surgery performed after the onset of sepsis and within 24 hr of ICU admission.

b Vasoactive-inotropic score = dopamine (µg/kg/min) + dobutamine (µg/kg/min) + 100 × epinephrine (µg/kg/min) + 100 × norepinephrine (µg/kg/min) + 10 × milrinone (µg/kg/min) + 10,000 × vasopressin (units/kg/min) + 50×levosimendan (µg/kg/min).

Data are presented as medians (interquartile ranges) or numbers (%). The *p* represents a comparative test between the PMX and non-PMX groups.

Complete patient characteristics between the two groups were contained in the Supplementary Table 1 (https://links.lww.com/CCX/B554), and a part of them were shown in Table 1. No significant differences were observed in age, APACHE II, and SOFA score between the groups. Although there was no difference in causative organisms between groups, the site of infection varied: urinary tract and abdominal infections were more common in the PMX group, whereas thoracic infections were more common in the non-PMX group (*p* = 0.031). Emergency surgeries were more prevalent, and the time from sepsis onset to antibiotics administration was shorter in the PMX group than in non-PMX group. No between-group differences were observed in the presence of DIC, MAP, or heart rate at admission; however, the median maximum dose of norepinephrine within 6 h of admission was higher in the PMX group than in the non-PMX group (0.30 [IQR, 0.24–0.43] vs. 0.28 [IQR, 0.20–0.40] µg/kg/min) (*p* = 0.016). In addition, the median maximum VIS within 6 hours of admission was higher in the PMX group than in the non-PMX group (38.2 [IQR, 28.4–49.8] vs. 33.0 (22.7–44.8)) (*p* = 0.01). The number of patients who received mechanical ventilation within 6 hours was similar between the groups (PMX group: 63 [76.8%], non-PMX group: 172 [75.8%], *p* = 0.85). However, CKRT was significantly higher in the PMX group than in the non-PMX group (51 [62.2%] vs. 56 [24.7%]; *p* < 0.001). Details of PMX-HP and the number of patients treated with PMX-HP by institutions are presented in **Supplementary Table 2** and **Supplementary Figure 1** (https://links.lww.com/CCX/B554). PMX-HP was administered to 82 (26.5%) patients. Among these, the first PMX-HP session began 265 minutes (IQR 113–480) after ICU admission and lasted for 1016 minutes (IQR 533–1359) (16.9 hr). Thirty-six patients (11.7%) underwent the second session for 1243 minutes (IQR 795–1488) (20.7 hr). Circuit coagulation occurred in 25 patients (30.5%) in the first session and 6 (16.7%) in the second session.

### Primary and Secondary Outcomes

**Figure 2.**
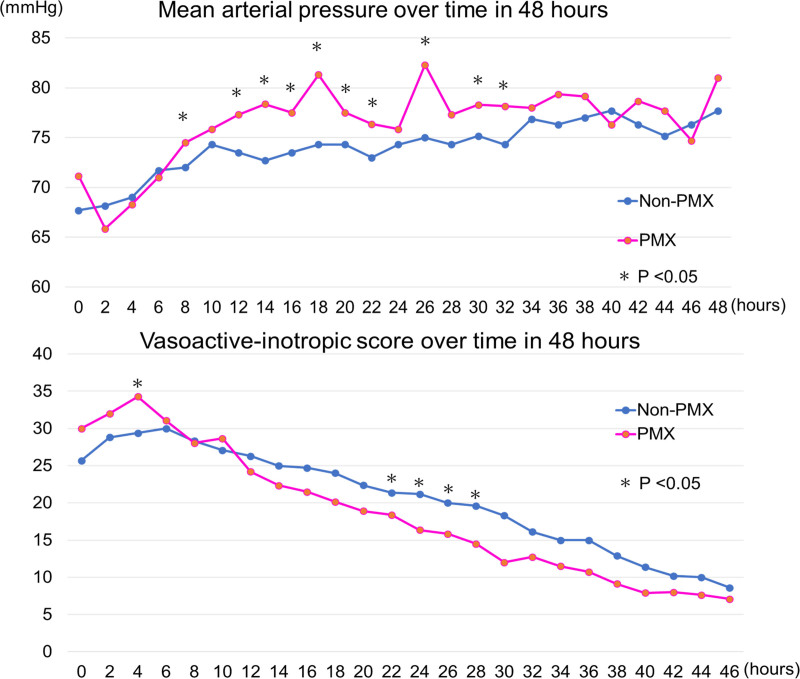
Blood pressure and vasoactive-inotropic score over time within 48 hours. The median of the data was plotted on the figure. *p values < 0.05. Vasoactive-inotrope score = norepinephrine (µg/kg/min) × 100 + epinephrine (µg/kg/min) × 100 + dopamine (µg/kg/min) + dobutamine (µg/kg/min) + vasopressin (unit/kg/min) × 10,000. PMX = polymyxin B.

**Table 2** and **Figure 2** present the primary and secondary outcomes. The 28-day mortality rate was similar between the two groups (PMX: 17.1%; non-PMX: 18.9%; *p* = 0.71). MAP was significantly higher from 8 to 32 hours after ICU admission in the PMX group than in the non-PMX group (Fig. 2). The maximum norepinephrine dose and VIS in the initial 6 h were higher in the PMX group than in the non-PMX group (Table 1); however, the VIS remained low at 12–46 hours (Fig. 2). The median number of 28-day ICU-free days was significantly lower in the PMX group than in the non-PMX group (PMX: 16 d [IQR, 0–20]; non-PMX: 18 d [IQR, 2–23]; *p* = 0.026) (Table 2).

**TABLE 2.** Primary and Secondary Outcomes

Outcomes	PMX Group (*n* = 82)	Non-PMX Group (*n* = 227)	*p*
28-d mortality	14 (17.1)	43 (18.9)	0.71
28-d ICU-free days	16 (0 to 20)	18 (2 to 23)	0.026
28-d ventilator-free days	20 (4 to 24)	22 (1 to 25)	0.36
28-d vasopressor-free days	22 (18 to 25)	23 (14 to 25)	0.73
28-d hemodialysis-free days	25 (14 to 28)	28 (16 to 28)	0.0071
Change in Sequential Organ Failure Assessment score in 3 d^a^	1 (–2 to 3)	0 (–3 to 2)	0.0025
Change in vasoactive-inotropic score in 48 hr^b^	–27.9 (–37.9 to –18.2)	–23.6 (–36 to –8)	0.087
Lactate clearance at 48 hr, %^c^	60.3 (38.8–74.6)	57.5 (37.5 to 73.8)	0.67
Fluid infusion during the first 48 hr, mL	8,149 (6,254 to 10,581)	6,264 (4,887 to 8,667)	< 0.0001
Transfusion within 48 hr			
RBC transfusion	36 (43.9)	71 (31.3)	0.039
Plasma transfusion	33 (40.2)	58 (25.6)	0.012
Platelet transfusion	24 (29.3)	49 (21.6)	0.16

Data are presented as medians (interquartile ranges) or *n* (%).

a Change in Sequential Organ Failure Assessment (SOFA) score in 3 days = (SOFA score at day 3—SOFA score at day 1)/SOFA score at day 1.

b Change in vasoactive-inotropic score (VIS) in 48 hr = VIS at 48 hr—maximum of VIS in initial 6 hr.

c Lactate clearance at 48 hr = lactate at ICU admission—lactate at 48 hr.

PMX = polymyxin B.

The change of SOFA score in the first 3 days was significantly worse in the PMX group compared with the non-PMX group (Table 2), and the coagulation sub-score of the SOFA score was significantly high at 48 hours after ICU admission (**Supplementary Table 3**, https://links.lww.com/CCX/B554).

The number of patients who received platelet transfusions within the first 3 days was similar between the groups. However, significantly more patients in the PMX group received RBC and plasma transfusions (43.9% vs. 31.3%; 40.2% vs. 25.6%, respectively) (Table 2). No difference was observed between the groups regarding changes in VIS and lactate clearance at 48 hours; however, fluid infusion volume during the first 48 hours was significantly higher in the PMX group than in the non-PMX group (Table 2).

**Figure 3.**
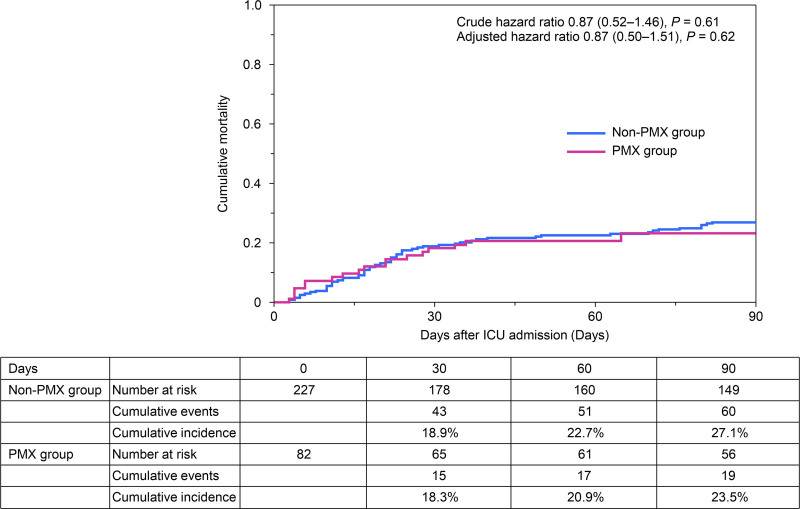
Cumulative mortality over 90 days. PMX = polymyxin B.

The 28-day and 90-day survival analyses showed no differences between the groups (adjusted HR [95% CI], 0.88 [0.46–1.68] for 28-d mortality, 0.87 [0.50–1.51] for 90-d mortality) (**Supplementary Fig. 2**, https://links.lww.com/CCX/B554; and **Fig. 3**).

### Subgroup Analyses

PMX-HP showed no statistically significant efficacy in any subgroups (**Supplementary Figs. 3** and **4**, https://links.lww.com/CCX/B554). The adjusted HRs [95% CIs] for 90-day mortality were 0.12 [0.015–0.93] (interaction *p* = 0.061) and 0.21 [0.052–0.86] (interaction *p* = 0.59) in the subgroups of SOFA score less than 11 and VIS less than 30, respectively; however, the interaction *p* value was not significant in both subgroups (Supplementary Fig. 3, https://links.lww.com/CCX/B554).

### Adverse Events

Thrombocytopenia (< 50,000/μL) within the first 3 days was significantly more common in the PMX group than in the non-PMX group (*p* = 0.048) (**Table 3**), despite a similar frequency of platelet transfusion (Table 2). Thrombocytopenia and platelet transfusion were not associated with the duration of PMX-HP (**Supplementary Table 4**, https://links.lww.com/CCX/B554) but appeared related to the implementation of PMX-HP or CKRT (**Supplementary Table 5**, https://links.lww.com/CCX/B554), with CKRT likely being the primary contributor to thrombocytopenia (**Supplementary Table 6**, https://links.lww.com/CCX/B554).

**TABLE 3.** Adverse Events

Adverse Events	All Patients (*n* = 309)	PMX Group (*n* = 82)	Non-PMX Group (*n* = 227)	*p*
New-onset arrhythmia within 48 hr	64 (20.7)	15 (18.3)	49 (21.6)	0.53
Thrombocytopenia (< 50,000/μL) within 48 hr	101 (32.7)	34 (41.5)	67 (29.5)	0.048
Leukopenia (< 3000/μL) within 48 hr	68 (22.0)	20 (24.4)	48 (21.2)	0.54

PMX = polymyxin B.

Data are reported as numbers (%).

## DISCUSSION

To our knowledge, this is the first study to explore the effects of PMX-HP on daily clinical courses using registry data from consecutive but exclusive patients with severe septic shock requiring greater than or equal to 0.2 µg/kg/min of norepinephrine. No associations were found between PMX-HP administration and 28-day or 90-day mortality. MAP was maintained at a higher level with low-dose vasopressor from 8 to 32 hours following admission in the PMX group, despite a higher norepinephrine dose and VIS at ICU admission. In this cohort of severe septic shock requiring high-dose norepinephrine, PMX-HP was performed for a considerably long duration of 1016 (533–1359) min for the first session and 1243 (759–1488) min for the second session.

PMX-HP is covered by insurance in Japan, allowing physicians to treat patients with presumable endotoxemic septic shock caused by gram-negative bacteria up to twice without measuring endotoxin levels. International and Japanese guidelines weakly recommended against its use (24, 25) owing to clinical study results in Europe and the United States (10, 12, 13), and its use remains controversial in Japan. In this observational study, an indication for PMX-HP was based on physician discretion and was not protocolized. Consequently, the frequency of usage of PMX-HP differed between institutions. The higher prevalence of urinary tract and abdominal infection, as well as the frequency of emergency surgeries in the PMX group, are consistent with this variability. Similarly, the shorter time to antibiotic administration observed in the PMX group may reflect differences in the infection site. Since early antibiotic administration is associated with improved outcomes in septic shock (26, 27), this factor may have contributed to a more favorable prognosis in the PMX group.

PMX-HP typically spans 2 hours outside Japan, whereas it may be performed for greater than 2 hours in Japan based on in vitro studies demonstrating endotoxin adsorption lasting up to 24 hours (28, 29). Consequently, PMX-HP was performed for an extended duration in this research group; however, this does not reflect the standard for Japanese physicians. The effect of PMX-HP on increasing blood pressure in septic shock has been reported previously (10, 11) and was evident in this study. High MAP was maintained from 8 to 32 hours following admission, and the VIS tended to be lower between 12 and 46 hours in the PMX group, suggesting that prolonged PMX-HP sustained the blood pressure-increasing effect. Elevated blood pressure in the PMX group did not translate into prognosis and may not indicate effective recovery. The MAP in the PMX group often exceeded 75 mm Hg, which might have been unnecessarily high in the early stages of septic shock. But earlier reduction in their use may potentially improve prognosis since high-dose catecholamines are associated with poor prognosis (30, 31). In addition, early administration of PMX-HP has been shown in several observational studies to be associated with improved hemodynamics and survival (32). The median time from ICU admission to administration of PMX-HP was 265 minutes in this study, and further study of the timing of administration of PMX-HP is required.

We did not observe an association between PMX-HP administration and survival improvement. The 28-day mortality rates were 17.1% and 18.9%, whereas the cumulative 90-day mortality rates were 23.5% and 27.1% in the PMX and non-PMX groups, respectively. In addition, the PMX group had significantly fewer 28-day ICU-free and hemodialysis-free days than those of the non-PMX group. Patients who received PMX-HP more commonly had abdominal and urinary tract infections, underwent more emergency surgeries, required higher doses of vasoactive-inotropic agents, and were more frequently treated with CKRT. In addition, they also received increased fluid volume and transfusion in the first 48 hours, with worsened SOFA scores over the subsequent 3 days. It remains unclear whether early deterioration in the clinical course was due to the patient’s baseline condition or the effects of PMX-HP. Given the potential risks, the use of PMX-HP should be considered only for carefully selected patients.

The coagulation sub-score of the SOFA score was significantly higher, and thrombocytopenia less than 50,000/μL was significantly more common after 48 hours in the PMX group. The number of patients who received platelet transfusions within the first 3 days was similar between the groups, but significantly more patients in the PMX group received RBC and plasma transfusions. PMX-HP is often performed for greater than 2 hours in Japan, with some durations extending up to 24 hours or until circuit coagulation, depending on the facility and physician. In Japan, PMX-HP is preferred to be greater than or equal to 6 hours to eliminate more endotoxins in critically ill patients based on previous findings that endotoxin adsorption by PMX-HP continues for at least 24 hours [19]. In this study, the durations of the first and second PMX-HP sessions durations notably long, with medians of 1016 minutes (533–1359) and 1243 minutes (759–1488), respectively. The extended duration may have contributed to a higher rate of circuit coagulation and increased consumption of blood cells and clotting factors. However, no clear association was observed between thrombocytopenia and the duration of PMX-HP.

Platelet consumption has been reported during KRT (33), increasing the risk of thrombocytopenia. Clinically, KRT is often performed concurrently with PMX-HP as part of blood purification therapy (34). In this study, a strong association between the use of PMX-HP and KRT was observed. Notably, thrombocytopenia was more closely associated with the implementation of CKRT rather than PMX-HP. In Japan, Nafamostat mesylate is commonly used for anticoagulation during both PMX-HP and CKRT, and no standardized anticoagulation protocols were used in this study. Therefore, it remains difficult to determine whether thrombocytopenia was influenced by anticoagulation management.

The subgroup analysis of 90-day prognosis implied a potential association between PMX-HP and a favorable HR in the subgroups with SOFA less than 11 and VIS less than 30, with a large interaction *p* value. These subgroup effects could be coincidental, considering the substantial interaction *p* value. However, given the concordant results of previous studies, PMX-HP may improve prognosis in patients without severe multiple organ failure or severe septic shock, which should be confirmed in future studies. A large Japanese registry study (15) reported an association between PMX-HP and a favorable prognosis in the group with SOFA scores of 7–12 but not in the group with more severe SOFA scores greater than 12 points. Similarly, the Euphrates trial in North America (10) demonstrated a significant mortality benefit of PMX-HP in patients with moderate elevations of Endotoxin Active Assay (EAA) between 0.6 and 0.9 but not in those with EAA greater than 0.9. An additional study on this moderately severe subgroup (the Tigris trial) is ongoing (35, 36). Thus, PMX-HP might be effective in patients with moderately severe sepsis.

This study has some limitations. First, this was an observational study rather than a randomized clinical trial; therefore, there are strong limitations as described below. Systematic differences exist inpatient backgrounds between the groups, suggestive of selection bias. In addition, an indication and methods for PMX-HP were not protocolized and were based on the physician's discretion. Furthermore, differences between facilities would affect the use of PMX-HP. Although we conducted adjusted and subgroup analyses using predefined factors to account for these biases, unmeasured residual confounders might still be present regardless of the selection of analytical models, due to the nature of the observational study design. Second, the overall mortality was significantly lower than the expected 40%, with 18% 28-day mortality and 25% 90-day mortality rates. Sepsis treatment outcomes may have improved in recent years (37). The small number of deaths could reduce the study’s power to detect clinically significant differences in outcomes and might result in overfitting in multivariate models using predefined adjustors. Third, the target sample size was 400; however, only 309 patients were enrolled, primarily attributed to a decrease in sepsis cases during the COVID-19 pandemic, which temporarily changed hospital functions and reduced research efforts. The smaller sample size weakened the study’s statistical power. Fourth, EAA or endotoxins were not measured; therefore, actual endotoxin adsorption was not evaluated. However, a clinically effective method for measuring endotoxin is not currently available; therefore, an alternative indicator, such as the initial VIS, is necessary to adopt PMX-HP. This study showed the possibility of identifying patients who might benefit from PMX-HP using clinically available variables other than EAA.

## CONCLUSIONS

PMX-HP was not associated with improved prognosis in patients with septic shock requiring norepinephrine, despite higher MAP in the early phase of ICU. These findings suggest potential disadvantages of PMX-HP, including increased fluid load and thrombocytopenia, underscoring the need for careful patient selection. Further randomized controlled trials are warranted to clarify the optimal indication and application methods for PMX-HP.

Dr. Kawazoe made substantial contributions to data acquisition and drafting of the article. Drs. Kawazoe, Miyamoto, and Morimoto contributed to the study design, statistical analysis, interpretation of the data, and drafting of the article. All other authors made substantial contributions to data acquisition and article review. Dr. Kawazoe critically revised the article for intellectual content. Dr. Morimoto supervised the study. All the authors have read and approved the final version of the article.

Consent for publication was not obtained from each patient, but the opt-out method disclosed that summarized data without individual identification would be published in the literature.

The datasets generated and/or analyzed in the present study are not publicly available owing to ongoing research; however, the data are available from the corresponding author upon reasonable request.

## ACKNOWLEDGMENTS

We extend our gratitude to the BEst Available Treatment for septic SHOCK Registry investigators and healthcare providers at the participating hospitals for their cooperation with this study. We would like to thank Atsushi Hiraide, Seiya Kato, and Hajime Furukawa for their contributions to the Data Monitoring Board, as well as Shigeki Kushimoto for his research advisor.

## Supplementary Material
