# Supplementary material for: Polymyxin B Hemoperfusion for Patients With Septic Shock Requiring High-Dose Norepinephrine: A Multicenter Prospective Cohort Study
Source: Crit Care Explor. 2025 Sep 22;7(10):e1320. doi: 10.1097/CCE.0000000000001320 (PMC12456497; doi:10.1097/CCE.0000000000001320)
Supplement: Supplementary file 1 [file cc9-7-e1320-s001.pdf]

Prolonged polymyxin B hemoperfusion for patients with septic shock requiring high-dose norepinephrine: a multicenter prospective cohort study

Additional files

Table of contents **Page**

Supplementary Table 1……………………………………………..………………..2
Supplementary Table 2……………………………………………..………………..4
Supplementary Figure 1…………………………………………………………………..5
Supplementary Table 3………………..……………………………………………..6
Supplementary Figure 2…………………………………………………………………..7
Supplementary Figure 3………………..…………………………………………………8
Supplementary Figure 4………………..…………………………………………………9
Supplementary Table 4 ..…………………………………………………………...10
Supplementary Table 5..………..…………………………………………………..11
Supplementary Table 6..………..…………………………………………………..12

Table 1. Complete patient characteristics and treatments upon intensive care unit admission

Characteristics and treatments	All patients (n = 309)	PMX group (n = 82)	Non-PMX group (n = 227)	*P*-value
Age (years)	72 (64–80.5)	71 (62–80)	73 (65–81)	0.26
Male sex	175 (56.6)	46 (56.1)	129 (56.8)	0.91
Body mass index (kg/m^2)	21.95 (19.5–25.6)	23.4 (20.7–27.6)	21.6 (19.3–24.8)	0.0028
APACHE II score	26 (21–32.5)	26 (21–31)	27 (22–33)	0.37
Comorbidity [a]				
Chronic hemodialysis	19 (6.1)	4 (4.9)	15 (6.6)	0.58
Immuno-compromised	15 (4.9)	4 (4.9)	11 (4.8)	1.00
Chronic respiratory disorder	6 (1.9)	3 (3.7)	3 (1.3)	0.19
Liver cirrhosis	5 (1.6)	0 (0)	5 (2.2)	0.33
Chronic heart failure	0 (0)	0 (0)	0 (0)	1
Charlson comorbidity index	1.0 (0–3.0)	1 (0–3)	1 (0–3)	0.29
Performance status prior to				0.10
hospitalization [b]	133 (43.0)	44 (53.7)	89 (39.2)	
Normal activity	81 (26.2)	21 (25.6)	60 (26.4)	
Restricted in strenuous activity	34 (11.0)	8 (9.8)	26 (11.5)	
Ambulatory but unable to carry out any work activities	35 (11.3)	6 (7.3)	29 (12.8)	
Stay in a chair or bed for more than 50% of the day	26 (8.4)	3 (3.7)	23 (10.1)	
Completely disabled and bedridden				
Site of infection				0.031
Abdomen	115 (37.2)	37 (45.1)	78 (34.4)	
Urinary tract	50 (16.1)	18 (22.0)	32 (14.1)	
Thorax	59 (19.1)	8 (9.8)	51 (22.5)	
Skin and soft tissue	56 (18.1)	14 (17.1)	42 (18.5)	

Others	29 (9.4)	5 (6.1)	24 (10.6)	
Bacteremia	159 (51.5)	46 (56.1)	113 (49.8)	0.33
Causative organisms				0.48
Gram-negative bacteria	135 (43.7)	39 (47.6)	96 (42.3)	
Gram-positive bacteria	50 (16.2)	10 (12.2)	40 (17.6)	
Others (including unknown)	124 (40.1)	33 (40.2)	91 (40.1)	
Lactate concentration (mmol/L)	4.1 (2.6–6.5)	4.2 (2.7–6.7)	4.0 (2.6–6.5)	0.78
SOFA score	11 (9–14)	11 (9–13)	11 (9–14)	0.093
Mean arterial pressure (mmHg)	69 (57–80)	71 (60–81)	68 (56–79)	0.11
Heart rate (bpm)	110 (93–124)	108 (90–127)	110 (95–124)	0.89
DIC [c]	162 (52.4)	38 (46.3)	124 (54.6)	0.20
Treatment				
Emergency surgery [d]	108 (35.0)	36 (43.9)	72 (31.7)	0.047
Time from sepsis onset to antibiotics administration, min	128 (67–240)	105 (60–179)	143 (70–262)	0.031
Inappropriate initial antibiotics [e]	8 (2.6)	0 (0)	8 (4.0)	0.12
Maximum dose of norepinephrine in 6 hrs (μg/kg/min)	0.30 (0.20–0.40)	0.30 (0.24–0.43)	0.28 (0.20–0.40)	0.016
Maximum VIS [f] in 6 hrs	34.4 (24.2–45.7)	38.2 (28.4–49.8)	33 (22.7–44.8)	0.012
Corticosteroids [g]	257 (83.2)	69 (84.1)	188 (82.8)	0.78
Invasive mechanical ventilation [g]	235 (76.1)	63 (76.8)	172 (75.8)	0.85
Continuous kidney replacement therapy [g]	107 (34.6)	51 (62.2)	56 (24.7)	<0.0001

Data are presented as medians (interquartile ranges) or numbers (%). The *P*-value represents a comparative test between the PMX and non-PMX groups.

[a] Comorbidity was defined in accordance with the APACHE II score definition.

[b] Performance status was classified according to the World Health Organization performance status classification.

[c] DIC was diagnosed using the JAAM-DIC diagnostic criteria.

[d] Emergency surgery was defined as surgery performed after the onset of sepsis and within 24 hours of ICU admission.

[e] Inappropriate initial antibiotics was defined as initial antibiotics to which the causative organisms were resistant.

[f] Vasoactive-inotropic score = dopamine (μg/kg/min) + dobutamine (μg/kg/min) + 100×epinephrine (μg/kg/min) + 100×norepinephrine (μg/kg/min) + 10×milrinone (μg/kg/min) + 10,000×vasopressin (units/kg/min) + 50×levosimendan (μg/kg/min).

[g] Corticosteroids, invasive mechanical ventilation and continuous kidney replacement therapy were started on the first or second calendar-days of ICU admission.

PMX: polymyxin-B; ICU: intensive care unit; SOFA: sequential organ failure assessment; APACHE II: Acute Physiology and Chronic Health Evaluation II; DIC: disseminated intravascular coagulation; VIS: vasoactive-inotropic score; JAAM: Japanese Association for Acute Medicine

Supplementary Table 2. Details of PMX-HP implementation

Details of PMX-HP implementation	First session (n = 82)	Second session (n = 36)
Time from ICU admission to PMX-HP (min)	265 (113–480)	-
Duration of PMX-HP (min)	1016 (533–1359)	1243 (795–1488)
Anticoagulation for hemoperfusion		
Nafamostat mesylate	76 (93)	30 (83)
Unfractionated heparin	5 (6)	5 (14)
None	1 (1)	1 (3)
Unplanned interruption of PMX-HP	31 (38)	8 (22)
Circuit coagulation	25 (31)	6 (17)
Other reasons	6 (7)	2 (5)

Data are reported as medians (interquartile ranges) or numbers (%).

PMX-HP; polymyxin-B hemoperfusion; ICU: intensive care unit

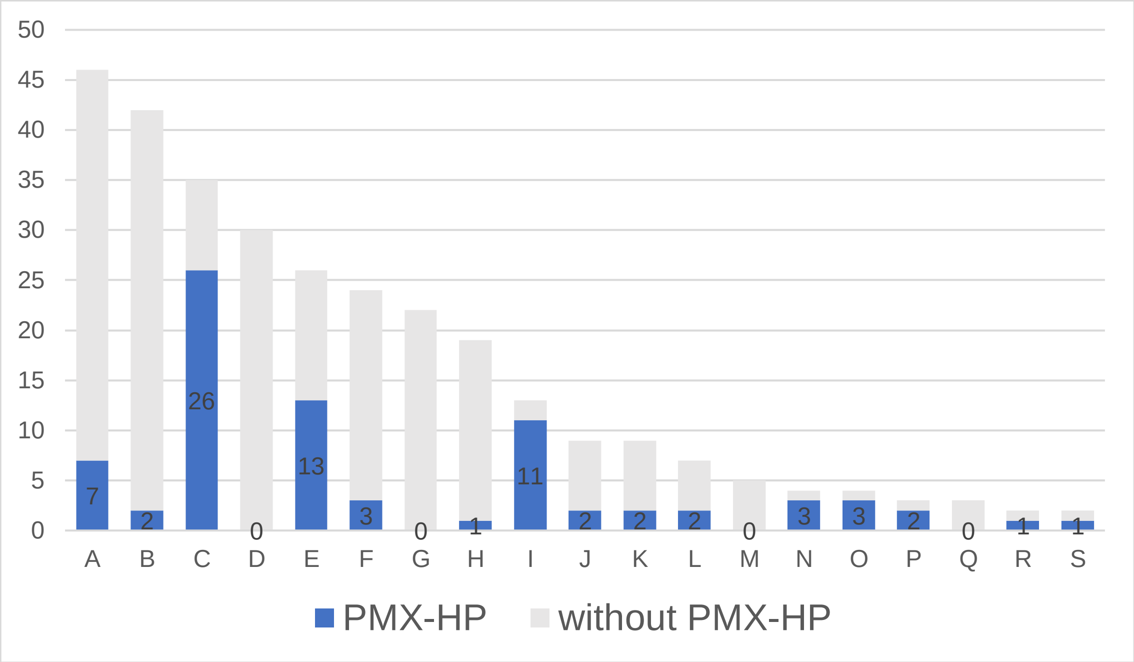

Supplementary Figure 1. Patients treated with PMX-HP by institutions

PMX-HP: plymyxin-B hemoperfusion

Supplementary Table 3. SOFA score on ICU admission and after 48 hours

Total and sub-score of SOFA score	All patients (n = 309)	PMX group (n = 82)	Non-PMX group (n = 227)	*P* value
On ICU admission				
Total SOFA score	11 (9–14)	11 (9–13)	11 (9–14)	0.15
Respiration sub-score	2 (1–3)	2 (0–2)	2 (1–3)	0.14
Coagulation sub-score	1 (0–2)	1 (0–2)	1 (0–2)	0.35
Liver sub-score	0 (0–1)	0 (0–1)	0 (0–1)	0.29
Cardiovascular sub-score	4 (4–4)	4 (4–4)	4 (4–4)	0.083
Central nervous system sub-score	2 (1–4)	1 (1–3)	2 (1–4)	0.066
Renal sub-score	2 (1–4)	2 (1–3)	2 (0–4)	0.76
After 48 hours				
Total SOFA score	11 (8–14)	12 (9–15)	11 (8–14)	0.16
Respiration sub-score	2 (1–3)	2 (1–2)	2 (1–2)	0.23
Coagulation sub-score	2 (1–3)	2 (2–3)	2 (1–3)	0.0095
Liver sub-score	0 (0–2)	0 (0–2)	0 (0–2)	0.89
Cardiovascular sub-score	4 (3–4)	3 (3–4)	4 (3–4)	0.12
Central nervous system sub-score	2 (1–3)	2 (1–3)	2 (1–3)	0.88
Renal sub-score	1 (0–3)	1 (0–4)	2 (0–3)	0.21

Data are reported as numbers (%).

PMX: polymyxin-B; ICU: intensive care unit; SOFA: sequential organ failure assessment

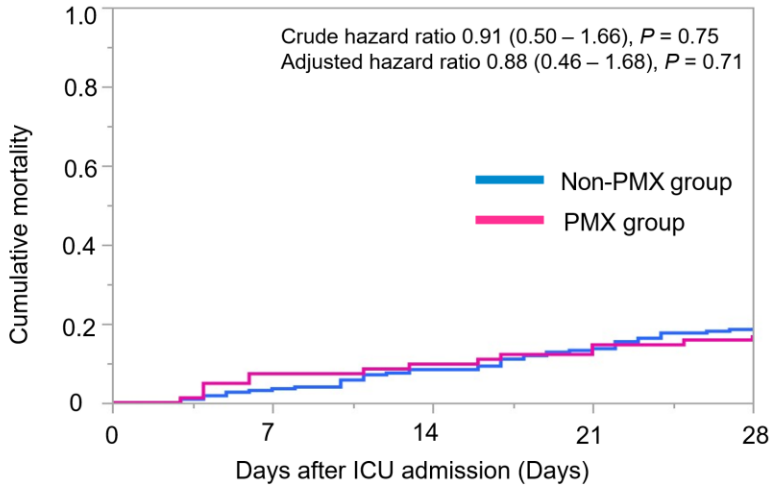

Days		0	7	14	21	28
no PMX group	Number at risk	227	219	208	196	184
	Cumulative events		8	19	31	43
	Cumulative incidence		3.5%	8.4%	13.7%	18.9%
PMX group	Number at risk	82	76	74	70	68
	Cumulative events		6	8	12	14
	Cumulative incidence		7.3%	9.8%	14.6%	17.1%

Supplementary Figure 2. Cumulative mortality over 28 days
PMX: polymyxin-B; ICU: intensive care unit

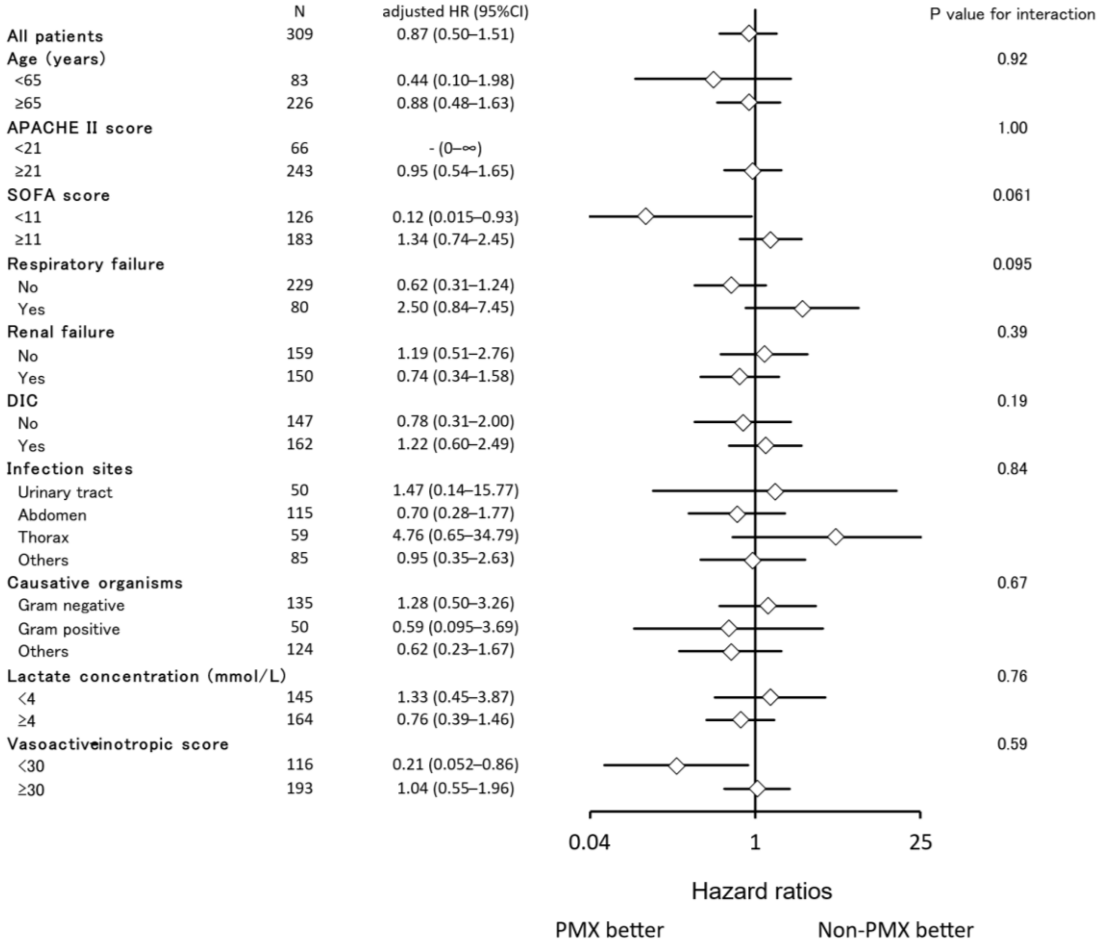

Supplementary Figure 3. Subgroup analyses of 90-day mortality using a multivariate Cox proportional hazard model

PMX: polymyxin-B; APACHE II: Acute Physiology and Chronic Health Evaluation II; SOFA: sequential organ failure assessment; DIC: disseminated intravascular coagulation; HR: hazard ratio; CI: confidence interval

	N	adjusted HR (95%CI)	P value for interaction
**All patients**	309	0.88 (0.46–1.68)	
**Age (years)**			0.48
<65	83	0.71 (0.15–3.49)	
≥65	226	0.77 (0.37–1.60)	
**APACHE II score**			1.00
<21	66	- (0–∞)	
≥21	243	0.95 (0.52–1.91)	
**SOFA score**			0.15
<11	126	0.17 (0.02–1.39)	
≥11	183	1.30 (0.65–2.61)	
**Respiratory failure**			0.58
No	229	0.76 (0.35–1.67)	
Yes	80	1.58 (0.42–5.97)	
**Renal failure**			0.93
No	159	0.96 (0.32–2.89)	
Yes	150	0.90 (0.39–2.06)	
**DIC**			0.44
No	147	0.95 (0.31–2.91)	
Yes	162	1.21 (0.53–2.74)	
**Infection sites**			0.32
Urinary tract	50	0.62 (0.34–11.36)	
Abdomen	115	0.52 (0.17–1.63)	
Thorax	59	3.23 (0.41–25.43)	
Others	85	0.98 (0.32–3.04)	
**Causative organisms**			0.46
Gram negative	135	1.23 (0.41–3.68)	
Gram positive	50	0.89 (0.13–5.89)	
Others	124	0.47 (0.13–1.65)	
**Lactate concentration (mmol/L)**			0.82
<4	145	1.13 (0.28–4.54)	
≥4	164	0.86 (0.40–1.82)	
**Vasoactive-inotropic score**			0.56
<30	116	0.22 (0.045–1.04)	
≥30	193	1.01 (0.48–2.13)	

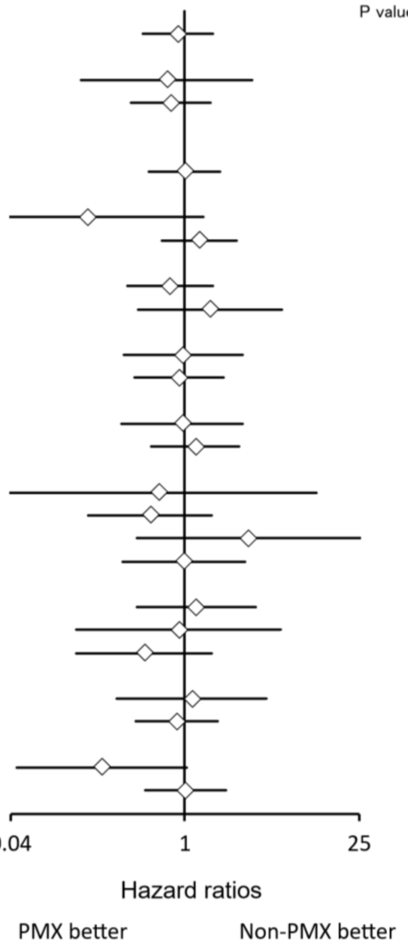

Supplementary Figure 4. Subgroup analyses of 28-day mortality using a multivariate Cox proportional hazard model

PMX: polymyxin-B; APACHE II: Acute Physiology and Chronic Health Evaluation II; SOFA: sequential organ failure assessment; DIC: disseminated intravascular coagulation; HR: hazard ratio; CI: confidence interval

Supplementary Table 4. PMX duration and adverse event

Total PMX duration, minutes	<715 (n = 19)	715<, 1372≥ (n = 22)	1372<, 2189≥ (n = 21)	2189< (n = 20)	*P*-value [a]
Transfusion within 48 h					
RBC transfusion	6 (31.6)	7 (31.8)	12 (57.1)	11 (55)	0.052
Plasma transfusion	9 (47.4)	8 (36.4)	9 (42.9)	7 (35)	0.55
Platelet transfusion	6 (31.6)	7 (31.8)	5 (23.8)	7 (35)	0.97
New-onset arrythmia within 48 hours	5 (26.3)	2 (9.1)	4 (19.1)	4 (20)	0.86
Thrombocytopenia (<50,000/μL) within 48 hours	10 (52.6)	7 (31.8)	9 (42.9)	8 (40)	0.62
Leukopenia (<3,000/μL) within 48 hours	4 (21.1)	5 (22.7)	5 (23.8)	6 (30)	0.52

Data are presented as numbers (%).

[a] P-value was obtained with Cochrane-Armitage trend test.

PMX: polymyxin-B hemoperfusion; RBC: red blood cell

Supplementary Table 5. Transfusion and thrombocytopenia in PMX and/or CKRT

PMX and/or CKRT	PMX (-), CKRT (-) (n = 171)	PMX (-), CKRT (+) (n = 25)	PMX (+), CKRT (-) (n = 31)	PMX (+), CKRT (+) (n = 51)	*P*-value
Transfusion within 48 h					
RBC transfusion	48 (28.1)	25 (44.6)	11 (35.5)	25 (49.0)	0.017
Plasma transfusion	39 (22.8)	21 (37.5)	11 (35.5)	22 (43.1)	0.016
Platelet transfusion	30 (17.5)	21 (37.5)	8 (25.8)	17 (33.3)	0.0083
Thrombocytopenia (<50,000/µL) within 48 hours	42 (24.6)	25 (44.6)	14 (45.2)	20 (39.2)	0.0076

Data are presented as numbers (%).

PMX: polymyxin-B hemoperfusion; CKRT: continuous kidney replacement therapy; RBC: red blood cell

Supplementary Table 6. Influence of PMX-HP and CKRT on thrombocytopenia

Variables	Odds ratio [a]	96%CIs	*P*-value [a]
Univariate analysis			
PMX-HP	1.69	1.00 - 2.85	0.051
CKRT	1.89	1.16 – 3.10	0.011
Multivariate analysis			
PMX-HP	1.39	0.79 – 2.43	0.25
CKRT	1.70	1.01 – 2.88	0.048

[a] Odds ratio was obtained with logistic regression model.

PMX-HP: polymyxin-B hemoperfusion; CKRT: continuous kidney replacement therapy
